# Adaptive dynamic resource allocation in annual eusocial insects: environmental variation will not necessarily promote graded control

**DOI:** 10.1186/1472-6785-7-16

**Published:** 2007-12-19

**Authors:** Oliver Mitesser, Norbert Weissel, Erhard Strohm, Hans-Joachim Poethke

**Affiliations:** 1 Field Station Fabrikschleichach, Universität Würzburg, Glashüttenstr. 5, D-96181 Rauhenebrach, Germany; 2 Biozentrum (Zoologie III), Universität Würzburg, Am Hubland, D-97074 Würzburg, Germany; 3 Institut für Zoologie, Universität Regensburg, Universitätsstr. 31, 93040 Regensburg, Germany

## Abstract

**Background:**

According to the classical model of Macevicz and Oster, annual eusocial insects should show a clear dichotomous "bang-bang" strategy of resource allocation; colony fitness is maximised when a period of pure colony growth (exclusive production of workers) is followed by a single reproductive period characterised by the exclusive production of sexuals. However, in several species graded investment strategies with a simultaneous production of workers and sexuals have been observed. Such deviations from the "bang-bang" strategy are usually interpreted as an adaptive (bet-hedging) response to environmental fluctuations such as variation in season length or food availability.

To generate predictions about the optimal investment pattern of insect colonies in fluctuating environments, we slightly modified Macevicz and Oster's classical model of annual colony dynamics and used a dynamic programming approach nested into a recurrence procedure for the solution of the stochastic optimal control problem.

**Results:**

1) The optimal switching time between pure colony growth and the exclusive production of sexuals decreases with increasing environmental variance. 2) Yet, for reasonable levels of environmental fluctuations no deviation from the typical bang-bang strategy is predicted. 3) Model calculations for the halictid bee *Lasioglossum malachurum *reveal that bet-hedging is not likely to be the reason for the graded allocation into sexuals versus workers observed in this species. 4) When environmental variance reaches a critical level our model predicts an abrupt change from dichotomous behaviour to graded allocation strategies, but the transition between colony growth and production of sexuals is not necessarily monotonic. Both, the critical level of environmental variance as well as the characteristic pattern of resource allocation strongly depend on the type of function used to describe environmental fluctuations.

**Conclusion:**

Up to now bet-hedging as an evolutionary response to variation in season length has been the main argument to explain field observations of graded resource allocation in annual eusocial insect species. However, our model shows that the effect of moderate fluctuations of environmental conditions does not select for deviation from the classical bang-bang strategy and that the evolution of graded allocation strategies can be triggered only by extreme fluctuations. Detailed quantitative observations on resource allocation in eusocial insects are needed to analyse the relevance of alternative explanations, e.g. logistic colony growth or reproductive conflict between queen and workers, for the evolution of graded allocation strategies.

## Background

The optimal allocation of accumulated resources to maintenance, growth, and reproduction is the central topic of life history theory. At any time during its life an organism must decide whether it will allocate available resources to maintenance, to somatic growth (that will allow for larger reproductive potential in the future), or to reproduction. In particular, the existence of a trade-off between growth and reproduction has been well confirmed [1,2]. Much theoretical and field work has been invested to understand the pattern of investment into growth and reproduction and to predict which allocation strategies will maximise an organism's fitness [3-5]. Since the first paper by Cole [6] theoretical analysis of life history strategies has focused on solitary organisms (for reviews see [1,2,4,7]). In contrast, since the eminent work of Macevicz and Oster [8] the evolutionary analysis of nest cycle dynamics in social species has not gained much further attention [9,10]. For social insects the problem of an optimised investment into growth and reproduction mostly concerns the growth of the colony as a whole; how much resources should be allocated to increase worker number and how much to the production of sexuals? As the answer to this problem strongly depends on the time left until the end of the season and as this quantity continuously changes we refer to optimal investment patterns as dynamic strategies. Dynamic allocation strategies in eusocial insects have first been analysed by Macevicz and Oster [8] and Oster and Wilson [11].

Macevicz and Oster [8] analysed the prototype of an annual eusocial colony cycle as exhibited by many vespid wasps, bumble bees and halictid bees and calculated optimal resource allocation strategies for the case of predictable or constant season length [8,12]. When season length is fixed and conditions are constant during the season the predicted optimal investment pattern is a simple "bang-bang" strategy with the annual productivity cycle divided into two phases; colonies should start with a phase of pure colony growth, i.e. the exclusive production of workers, and – at some time – abruptly switch to a purely reproductive phase with the exclusive production of male and female sexuals. The optimal moment to switch between the two phases is entirely determined by season length, worker productivity rate, and worker mortality rate.

However, already Greene [13] has pointed out that colony development of many annual eusocial insects does not conform to the predicted bang-bang strategy but is characterised by a gradual shift from the production of workers to the production of sexuals. Such "graded control" has been reported in wasps [13-19], bumble bees [20,21] and halictids [22-25].

Although sufficiently detailed quantitative data are hardly available, the halictid bee *Lasioglossum malachurum *can serve as one example of this type of colony dynamics. Recent studies of *L. malachurum *around Wuerzburg (northern Bavaria, Germany) provide day-by-day observations of the timing of reproduction in five colonies [24,26]. These data clearly demonstrate the existence of graded control in this species and allow quantifying the length of the transition phase, when there is a simultaneous production of workers and sexuals.

Graded resource allocation strategies like that observed in *L. malachurum *are often interpreted as an evolutionary, risk spreading response (bet-hedging) to environmental stochasticity [1,2]. If the complete population under consideration suffers from identical but unpredictable year to year variation in productivity, mortality, or season length we expect bet-hedging strategies to be favoured by natural selection [27,28]. For example, in response to fluctuating season length plants may produce offspring with different diapause strategies [27,29]. Crickets may produce micropterous as well as macropterous individuals in response to variable availability of annual thermal energy [30], and young mice and voles may vary in age of maturity within populations in dynamic environments [31]. For solitary insects Hopper [32] has reviewed numerous cases where the occurrence of mixed strategies has been linked to the spreading of risk.

Oster and Wilson (1978) were the first to apply the general argument of bet-hedging to colony dynamics. They suggested that it could be the ultimate mechanism responsible for the evolution of graded control in social insects: "It can be demonstrated that stochastic variation in the system parameters will always promote graded control [11]." Following Oster (Fig. 2.16 in [11]) a risk spreading investment strategy in eusocial insects would be realised as a gradual sigmoid (instead of a dichotomous) transition between worker and sexual production. As a correlation between variation in season length (as a specific and common example of environmental fluctuations) and graded strategies is supported by many studies [30,33,34], variation in season length is often invoked to explain the occurrence of graded control in annual eusocial insects [9,11,17,22,23].

Hopper [32] reviewed several (potential) strategies of risk-spreading in solitary organisms: temporal, metapopulation, and within-generation spreading of risk. He concludes that the empirical evidence in support of bet-hedging as an important driver for the evolution of facultative diapause, migration polyphenism, spatial distribution of oviposition, egg size, and other traits is weak or doubtful, and that inter-annual environmental variability often turns out to be too weak to favour (substantial) risk-spreading. As the plausible verbal arguments of Oster and Wilson [11] in favour of bet hedging as the ultimate cause for graded control have never been worked out in detail, it remains an open question as to whether environmental fluctuations are sufficient to explain the evolution of graded investment strategies in eusocial insects.

In this paper we present a formal analysis of the influence of environmental stochasticity (implemented as fluctuations in season length) on the investment strategy of social insects. Based on the colony model of Macevicz and Oster [8] we investigate how the optimal strategy of temporal resource allocation is influenced by the distribution of environmental conditions (mean, variance and shape of the distribution of expected season length). For the special case of *Lasioglossum malachurum *we derive estimates of the variability of seasons. They allow us to predict optimal temporal resource allocation for *L. malachurum *and to check whether environmental fluctuations can explain the broad transition phase between colony growth and reproduction sometimes observed in this species.

## Results

### Deterministic environments

Our analysis is organised in two steps. First, we present a deterministic model of the colony cycle with constant season length. As numerical optimisation methods are required later and the nocturnal inactivity of the colonies provides a natural time base, we use a time-discrete version of the classical model of Macevicz and Oster [8] with a time step of one day. Secondly, we calculate the optimal investment strategy when season length varies according to a given distribution.

Our model represents colony development during a single season of length *L *(this condition will be relaxed later). Two main dependent variables describe the state of a colony: the number of workers (*W*_*i*_) and the number of sexuals (*S*_*i*_) at time step *i*. The colony cycle typically starts in spring with nest founding by inseminated and hibernated females. During the founding phase the females work alone and perform all those foraging tasks that will be taken over by workers after their emergence later in the season [12]. Thus, we start with initial condition *W*_1 _= 1 assuming that the founding queen acts like a single worker until the first eggs have developed to adults [35]. The change in the number of nestmates is governed by two mechanisms: mortality and reproduction. Each individual survives from time step *i *to *i *+ 1 with probability *q*. Resource allocation in each time step (*i*) is directly proportional to the current worker force (*W*_*i*_). Each worker can provision *c *(worker efficiency) eggs (= brood cells) per time step. For the sake of simplicity survival and efficiency of individuals are assumed to be constant during the whole season. We further assume that the actual egg laying rate of the queen is not limited, but the number of eggs that can be successfully provisioned depends (linearly) on the number of workers in the colony [35]. A time dependent fraction (*u*_*i*_) of resources is allocated to the production of sexuals while the fraction (1-*u*_*i*_) is invested into the production of workers. Thus, the number of workers (*W*_*i *+ 1_) at time step *i *+ 1 can be calculated as

*W*_*i *+ 1 _= *q*_*i*_*W*_*i *_+ *(*1 - *u*_*i*_*) c*_*i*_*W*_*i *_

In most eusocial halictid bees, annual vespid wasps, and bumble bees the life span of queens is much longer than life span of workers [12,36]. Female sexuals have to hibernate before nest founding in the following year, while workers live only for several weeks. Thus, we neglect mortality of sexuals as has been done by Macevicz and Oster [8] in most of their analyses. Consequently the number of sexuals (*S*_*i *+ 1_) at time step *i *+ 1 can be calculated as

*S*_*i *+ 1 _= *S*_*i *_+ *u*_*i*_*c*_*i*_*W*_*i *_

Like Macevicz and Oster we do not differentiate between investment in male and female sexuals. Thus *S*_*i *_is the total investment in both sexes and as the cost of male and female sexuals differs significantly in halictids [24] the total number of sexuals will depend on the sex ratio produced by the colony. As we do not want to complicate the paper by including sex ratio considerations we will in the following – according to Macevicz and Oster – simply call S the number of sexuals.

These two equations fully determine the development of an annual primitively eusocial bee colony from nest founding at time step *i *= 0 until the end of the season (*i = L*). The fitness of colonies following such nest dynamics can be measured by the final number of sexuals successfully raised (*S*_*L*_). Macevicz and Oster [8] as well as Oster and Wilson [11] have studied such systems (in time continuous form) as optimal control problems with control variable *u*_*i *_(fraction of resources allocated to the production of sexuals). In the deterministic case (when season length *L *does not change between years) they found that the (time-dependent) optimal control solution (*u*_*i*_) that maximises *S*_*L *_is a dichotomous bang-bang strategy and *u*_*i *_should switch from 0 to 1 at an optimal switching time (*SWT*). Thus, the optimal temporal pattern of reproduction consists of two distinct phases, a growth phase with exclusive worker production (*u*_*i *_= 0 for *1 ≤ i *<*SWT*) followed by a reproductive phase characterised by the exclusive production of sexuals (*u*_*i *_= 1 for *SWT *<*i ≤ L*). Accordingly, the optimal strategy can be characterised by a single parameter, the optimal switching time *SWT ∈*{1, 2, ..., *L*}, when sexual reproduction begins and *u*_*i *_(*i ∈ *{1, 2, ..., *L*}) changes from 0 to 1.

The switching time *SWT *can easily be calculated numerically. Just generate *L *different sequences *u*= {*u*_1 _= 0, *u*_2 _= 0, ..., *u*_*SWT *_= 0, *u*_*SWT *+ 1 _= 1, ..., *u*_*L *_= 1} (corresponding to different *SWTs*), iterate eqns. 1 and 2 for each *u*, and choose that *u*sequence (and corresponding *SWT*) that maximises colony fitness S_*L*_. In fact, the optimal *SWT *can also be found analytically in the case of time discrete dynamics, but cannot be computed by the formula provided by Macevicz and Oster [8] for the case of time-continuous dynamics (Mitesser, unpublished). The optimal *SWT *increases with increasing survival *q *and increasing worker efficiency *c *(see also eqn. 6 and Fig. 8 in [8]). The growth phase of the resulting colony dynamics is characterised by an exponential increase in worker number while the number of sexuals stays at 0 until the optimal *SWT *is reached. After that, worker number exponentially declines and the number of sexuals exponentially increases.

### Environmental stochasticity

Coarse grained environmental stochasticity (sensu [37]) could affect the model system in several different ways; worker survival rate, worker productivity rate as well as season length can change from year to year. Here we restrict our analysis to the presumably most common effect in the context of bet-hedging; variation in season length [30,32,38,39]. Variation in season length might be caused by differences in both, the beginning and the end of the season. However, to model variation in season length it is sufficient to change the number of time steps *L *available within a single year, as allocation strategies always refer to the time passed since colony foundation.

To analyse the optimal investment strategy in variable environments a few essential modifications of the model system have to be made. As season length *L*_*j *_varies between years *j*, reproductive output *S*_*Lj *_will also vary and different years will contribute differently to overall fitness. As all colonies of a population simultaneously suffer from identical (and unpredictable) environmental fluctuations, the appropriate measure of overall fitness (*F*) of the strategy is the *geometric *mean of single year reproductive output [1,2,37]. We use the frequency distribution *f(L) *to describe the distribution of season lengths (*L*). Thus, each single year reproductive output *S*_*Lj *_must be weighted according to the frequency *x*_*j *_= *f*(*L*_*j*_) of the corresponding season length *L*_*j*_. For the expected long term fitness of a strategy we get

F=(∏j=1nSLjxj)1/n
 MathType@MTEF@5@5@+=feaagaart1ev2aaatCvAUfKttLearuWrP9MDH5MBPbIqV92AaeXatLxBI9gBaebbnrfifHhDYfgasaacPC6xNi=xI8qiVKYPFjYdHaVhbbf9v8qqaqFr0xc9vqFj0dXdbba91qpepeI8k8fiI+fsY=rqGqVepae9pg0db9vqaiVgFr0xfr=xfr=xc9adbaqaaeGacaGaaiaabeqaaeqabiWaaaGcbaGaemOrayKaeyypa0JaeiikaGYaaebCaeaacqWGtbWudaqhaaWcbaGaemitaW0aaSbaaWqaaiabdQgaQbqabaaaleaacqWG4baEdaWgaaadbaGaemOAaOgabeaaaaaaleaacqWGQbGAcqGH9aqpcqaIXaqmaeaacqWGUbGBa0Gaey4dIunakiabcMcaPmaaCaaaleqabaGaeGymaeJaei4la8IaemOBa4gaaaaa@415E@

Bet-hedging analyses have been based on various assumptions about the shape of the frequency distribution of environmental quality. However, season length *L *= 0 must always be excluded from the distribution of possible seasons (*f*(0) = 0) [30], otherwise vanishing fitness in a single year with length *L *= 0 would imply that mean fitness *F *equals 0, whatever the shape of the distribution is like. Thus, every time discrete model must assume a minimum season length of at least one time step. Apart from this, distributions representing environmental fluctuations in modelling approaches may vary from uniform distributions ([33,40], typically characterised by their lower und upper boundary) to normal distributions ([30], characterised by mean and variance). Thus, we investigated optimal allocation strategies for uniform and (approximately) normal distributions as representatives of two extreme frequency distributions, assuming that natural distributions fall somewhere in between.

As we use a time-discrete model system, season length *L*_*j *_in year *j *may only take integer values, while the normal distribution applies to continuous values. If season length is normally distributed (as assumed by [30]) with mean season length *μ *and variance *σ*^2^, then the exponent *x*_j _can be calculated as follows:

xj=∫Lj−12Lj+1212πσe−(L−μ)22σ2dL
MathType@MTEF@5@5@+=feaagaart1ev2aaatCvAUfKttLearuWrP9MDH5MBPbIqV92AaeXatLxBI9gBaebbnrfifHhDYfgasaacPC6xNi=xI8qiVKYPFjYdHaVhbbf9v8qqaqFr0xc9vqFj0dXdbba91qpepeI8k8fiI+fsY=rqGqVepae9pg0db9vqaiVgFr0xfr=xfr=xc9adbaqaaeGacaGaaiaabeqaaeqabiWaaaGcbaGaemiEaG3aaSbaaSqaaiabdQgaQbqabaGccqGH9aqpdaWdXbqaaKqbaoaalaaabaGaeGymaedabaWaaOaaaeaacqaIYaGmiiGacqWFapaCaeqaaiab=n8aZbaakiabdwgaLnaaCaaaleqabaGaeyOeI0scfa4aaSaaaeaacqGGOaakcqWGmbatcqGHsislcqWF8oqBcqGGPaqkdaahaaqabeaacqaIYaGmaaaabaGaeGOmaiJae83Wdm3aaWbaaeqabaGaeGOmaidaaaaaaaGccqWGKbazcqWGmbataSqaaiabdYeamnaaBaaameaacqWGQbGAaeqaaSGaeyOeI0scfa4aaSGaaeaacqaIXaqmaeaacqaIYaGmaaaaleaacqWGmbatdaWgaaadbaGaemOAaOgabeaaliabgUcaRKqbaoaaliaabaGaeGymaedabaGaeGOmaidaaaqdcqGHRiI8aaaa@54D2@

If season length is distributed uniformly between *B *- *μ *and *B *+ *μ *[33] with mean season length *μ *and width *B*, then *x*_*j *_does not depend on *j *(for *B *- *μ *<*j *<*B *+ *μ*) and can be calculated as follows:

xj={12Bfor B−μ<j<B+μ0otherwise
 MathType@MTEF@5@5@+=feaagaart1ev2aaatCvAUfKttLearuWrP9MDH5MBPbIqV92AaeXatLxBI9gBaebbnrfifHhDYfgasaacPC6xNi=xI8qiVKYPFjYdHaVhbbf9v8qqaqFr0xc9vqFj0dXdbba91qpepeI8k8fiI+fsY=rqGqVepae9pg0db9vqaiVgFr0xfr=xfr=xc9adbaqaaeGacaGaaiaabeqaaeqabiWaaaGcbaGaemiEaG3aaSbaaSqaaiabdQgaQbqabaGccqGH9aqpdaGabaqaauaabaqaciaaaKqbagaadaWccaqaaiabigdaXaqaaiabikdaYiabdkeacbaaaOqaaGqaaiab=zgaMjab=9gaVjab=jhaYjabbccaGiabdkeacjabgkHiTGGaciab+X7aTjabgYda8iabdQgaQjabgYda8iabdkeacjabgUcaRiab+X7aTbqaaiabicdaWaqaaiab=9gaVjab=rha0jab=HgaOjab=vgaLjab=jhaYjab=Dha3jab=LgaPjab=nhaZjab=vgaLbaaaiaawUhaaaaa@51EB@

To compare the effect of normally and uniformly distributed season length on the optimal strategy we characterised both distributions by their variance; for the uniform distribution with width *B *it always is σ2=B23
 MathType@MTEF@5@5@+=feaagaart1ev2aaatCvAUfKttLearuWrP9MDH5MBPbIqV92AaeXatLxBI9gBaebbnrfifHhDYfgasaacPC6xNi=xH8viVGI8Gi=hEeeu0xXdbba9frFj0xb9qqpG0dXdb9aspeI8k8fiI+fsY=rqGqVepae9pg0db9vqaiVgFr0xfr=xfr=xc9adbaqaaeGacaGaaiaabeqaaeqabiWaaaGcbaacciGae83Wdm3aaWbaaSqabeaacqaIYaGmaaGccqGH9aqpjuaGdaWccaqaaiabdkeacnaaCaaabeqaaiabikdaYaaaaeaacqaIZaWmaaaaaa@3381@

In the following, graded strategies will be characterised by the width *w *of the transition zone, i.e. the number of time steps for which the optimal *u*_*i *_surpasses a value of 0.05 but remains below 0.95. This is an appropriate measure as *u*_*i *_usually increases monotonically over time (exceptions to this rule will be discussed later).

For stochastic environments with variable season length (*L*) the control function *u*_*i *_(*i *= 0, ..., *L*) maximising fitness (*F*) cannot be calculated in the same straight forward way as before. In general, a recurrence method is required (see section Methods). Numerical results were calculated with the computer algebra system Mathematica 4.0 [41] and figures were plotted with the programming language R [42].

### Estimating model parameters

To quantify the optimal allocation strategies we must estimate the relevant parameters for both, colony dynamics and environmental conditions. *Lasioglossum malachurum *is the best studied social halictid in Europe, where it shows enormous clinal variation in its social behaviour [35]. In its northern range it produces only a single worker brood and then a brood of sexuals, whereas in southern Europe it produces as many as three worker broods and a final gyne brood. Field observations of the halictid bee *L. malachurum *around Wuerzburg provide the most comprehensive data on colony development. In Wuerzburg a typical (foraging) season lasts about 80 to 120 days [26]. However, the nest cycle is organised in a sequence of active and inactive periods [10], and only during approximately half of this time foraging and provisioning is observed [26]. Thus, we assume a mean season length of *L *= 50 foraging days. Mean worker life-time during foraging is about three weeks and survival rate per day can be approximated by *q *= 0.95 [35]. Worker life-time efficiency in *L. malachurum *is about 3.2 offspring per worker [24,43]. In combination with the survival rate this results in a worker productivity rate of 3.2/21 ≈ 0.15. These values predict an overall production of about 40 sexuals, a typical value for *L. malachurum *colonies at Wuerzburg [24,35,44].

Variation in season length can only be estimated on a rather coarse level. Data of the duration of the yearly number of foraging days in halictids are only available for a few years (Weissel, unpublished). Thus, we based our calculations on numerous correlates of season length of halictid colonies. The length of the activity period is influenced by several factors with temperature (in particular soil temperature that influences the rate of brood development [26]), presumably being the most important. Furthermore, the availability of flowers for harvesting nectar and pollen might affect the duration of the flight season [45]. As direct measures of these variables are not available, we calculated the coefficient of variation for six possible indicators of season length based on daily temperatures and phenological data on the annual vegetation cycle provided by the German weather service (Deutscher Wetterdienst) since 1947: available cumulative degree days [d°C] above the zero development temperature of 10.5°C of *L. malachurum *(Weissel, unpublished), the number of days with mean temperature above 10.5°C, the time span between first flowering of Anemona (*Anemona nemorosa*) and first fruits of Common oaks (*Quercus robur*), the time span between first flowering of Anemona and grape gathering (Mueller-Thurgau), the time span between first flowering of Coltsfoot (*Tussilago farfara*) and first fruits of Common oaks, and the time span between first flowering of Coltsfoot and grape gathering. Anemona and Coltsfoot flower around the time of nest founding in *L. malachurum *and oak fruiting as well as grape gathering occur around the end of the emergence period of *L. malachurum*. There might be no perfect correlation of raw temperature data and flowering dates of plants with the activity period of bees. Nevertheless, there is probably a sufficient statistical concordance to warrant the use of these indicators to estimate variation in season length.

**Figure 1:**
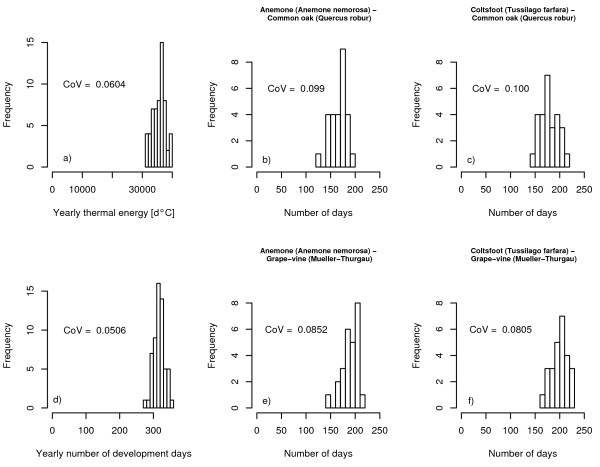
Frequency distribution of different indicators of season length: the yearly temperature sum [d°C] above the zero development temperature of 10.5°C of *L. malachurum *(Weissel, unpublished): *n *= 59 years (a), the time span between first flowering of Anemona (*Anemona nemorosa*) and first fruits of Common oaks (*Quercus robur*) : *n *= 27 years (b), the time span between first flowering of Coltsfoot (*Tussilago farfara*) and first fruits of Common oaks: *n *= 27 years (c), the number of days with mean temperature above 10.5°C: *n *= 59 years (d), the time span between first flowering of Anemona and grape gathering (Mueller-Thurgau) : *n *= 26 years (e), and the time span between first flowering of Coltsfoot and grape gathering: *n *= 26 years (f).

All estimates of the variability of season length for *L. malachurum *yielded similar values (Fig. 1) lending some confidence in the validity of these estimates. Thermal energy available for brood development typically varies by about 5% from year to year (Fig. 1a and 1d). Variation with regard to the dates of flowering of certain plants is less than 10% for all data sets (Fig. 1b, c, e, and 1f). If we use the maximum coefficient of variation (10% for the time span between first flowering of Coltsfoot (*Tussilago farfara*) and first fruits of Common oaks) observed in these data sets we would expect a typical standard deviation in the length of the foraging season of about 5 days. This is consistent with our observations of the colony activities of *L. malachurum *in the years 2002, 2003 and 2004 (Weissel, unpublished).

### Numerical results

To analyse the general behaviour of the model system we will first consider the uniform distribution of season lengths [33]. From this simple case we will proceed to the normal distribution [30] and subsequently discuss the different results.

**Figure 2:**
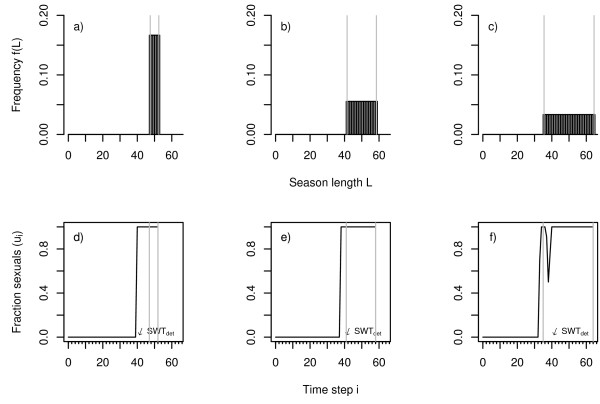
Uniformly distributed season length and corresponding strategy response. The upper row shows the frequency distribution of season length for three cases with increasing variance (a: width *B *= 5, b: *B *= 10, and c: *B *= 15, mean *μ *always = 50). The lower row shows the corresponding optimal strategy transitions: the fraction of sexuals produced by the colony as a function of time (d, e, and f, worker productivity rate *c *= 0.15, survival rate *q *= 0.95). A small arrow indicates the temporal position of the optimal switching point in the case of a deterministic environment (*SWT*_*det*_). Vertical grey lines indicate the boundaries of the distribution of season length. Figures d and e demonstrate that the optimal response of the system to increasing variation in season length is initially realised by an earlier switching from worker to sexual production. Graded strategies only emerge if environmental variation reaches a certain level and earlier switching alone would not be sufficient to buffer environmental fluctuations (c and f).

**Figure 3:**
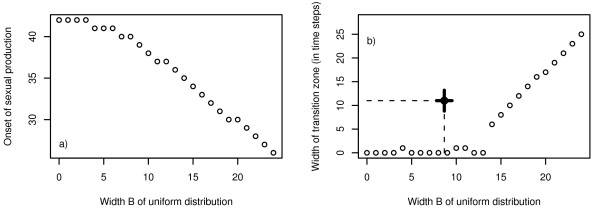
Onset of sexual production (a) and width *w *of the transition zone (b) between complete worker and complete sexual production as a function of the width of a uniform distribution of season length (*B*). *w *is defined as the number of time steps between the time when the strategy variable *u*_*i *_surpasses a value of 0.05 for the first time and the time when *u*_*i *_has finally reached at least 0.95 (and stays above this value for all remaining time steps). As long as *w *= 0, the reproduction strategy is bang-bang (as in the deterministic case), but the optimal switching time moves to earlier points in time when variance increases (a). Model parameters: worker productivity rate *c *= 0.15, survival rate *q *= 0.95, mean season length *μ *= 50. The single emphasised point in the right figure denotes the combination of strategy transition and estimation of environmental variance observed for *L. malachurum *at Wuerzburg. The bars indicate the 95% confidence interval for the estimation of the mean *w *(between colonies, n_1 _= 5 colonies) and the estimation of *B *(between years, n_2 _= 48).

The optimal response of the model system to (uniformly distributed) fluctuating season length consists of two consecutive phases: 1) for low to moderate fluctuations of season length (Fig. 2a and 2b) the typical bang-bang strategy with an abrupt transition between worker and sexual production is optimal. With increasing environmental variance the temporal position of the optimal switching point between growth and reproduction decreases (Fig. 2d and 2e) but – in contrast to Oster & Wilson's prediction [11] – the typical bang-bang strategy performs better than a gradual change from worker to sexual production. 2) When variance in season length exceeds a critical level (Fig. 2c) the bang-bang strategy is no longer adequate and a graded resource allocation strategy with an intermediate period of simultaneous production of workers and sexuals (and a humped strategy transition) becomes optimal (Fig. 2f). Graded resource allocation is characterised by a distinct phase of simultaneous production of workers and sexuals. It is a bet hedging strategy to avoid complete colony failure (Fig. 2f). With increasing variance of season length colonies start to reproduce earlier in their life cycle. However, while this temporal shift of the onset of reproduction is continuous (Fig. 3a), the transition between a pure bang-bang strategy and graded resource allocation is predicted to be rather abrupt (Fig. 3b). Figure 3b also provides field data on the transition zone observed in *L. malachurum *at Wuerzburg, which is obviously not in accordance with model predictions. This important aspect is presented in detail at the end of this section.

Further, the transition between colony growth and reproduction is not characterised by a monotonous increase in the amount of resources allocated to reproduction as suggested by Oster & Wilson (see Fig. 2.16 in [11]). Instead, the onset of the transition phase is characterised by a short pulse of nearly exclusive sexual production followed by a phase of simultaneous production of workers and sexuals before the colony finally ends with the exclusive production of sexuals. With increasing variance in season length the transition zone between pure colony growth and pure reproduction gets broader and the hump of worker production at the beginning of this zone becomes more pronounced. Tests with more restricted strategy sets (not shown here, see discussion) showed that this humped transition zone in fact significantly increases fitness compared to a monotonous sigmoid transition.

**Figure 4:**
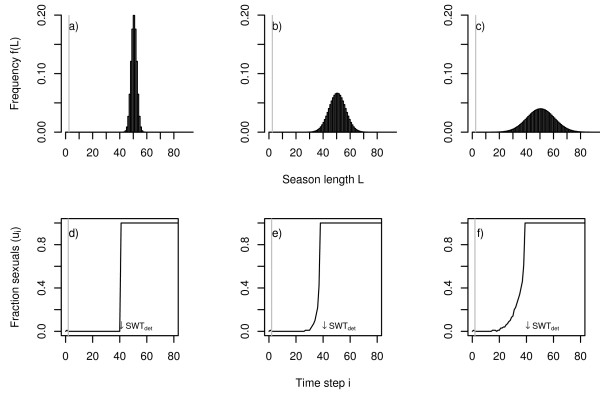
Normally distributed variation in season length and corresponding strategy response. The upper row shows the frequency distribution of season length for three cases with increasing variance (a: width standard deviation *σ *= 2.9, b: *σ *= 5.8, and c: *σ *= 8.7, mean *μ *always = 50). The lower row shows the corresponding optimal strategy transitions: the fraction of sexuals produced by the colony as a function of time (d, e, and f, worker productivity rate *c *= 0.15, survival rate *q *= 0.95). A small arrow indicates the temporal position of the optimal switching point in the case of a deterministic environment (*SWT*_*det*_). The vertical grey lines indicate the lower boundary of the distribution of season length at *L *= 1. The optimal response of the system to increasing variation in season length is initially realised solely by switching earlier from worker to sexual production, and graded strategies are realised when environmental variation increases (e, f).

**Figure 5:**
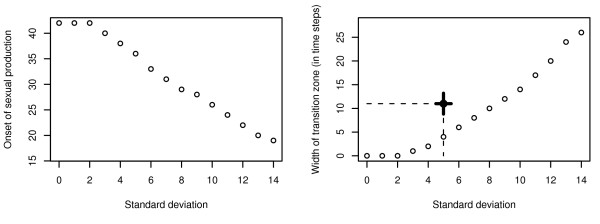
Onset of sexual production (a) and width *w *of the transition zone between complete worker and complete sexual production (b) as a function of the width of a normal distribution of season length (given as the standard deviation). *w *is defined as the number of time steps between the time when the strategy variable *u*_*i *_surpasses a value of 0.05 for the first time and the time when *u*_*i *_has finally reached at least 0.95 (and stays above this value for all remaining time steps). As long as *w *= 0 (see b), the reproduction strategy is bang-bang (as in the deterministic case), but the optimal switching time moves to earlier points in time when variance increases (a). Model parameters: worker productivity rate *c *= 0.15, survival rate *q *= 0.95, mean season length = 50. The single emphasised point in the right figure denotes the combination of strategy transition and estimation of environmental variance observed for *L. malachurum*. The bars indicate the 95% confidence interval for the estimation of the mean *w *(between colonies, n_1 _= 5 colonies) and the estimation of *B *(between years, n_2 _= 48).

The pattern described above clearly depends on the specific form of the distribution of season length. When the uniform distribution is replaced by a normal distribution, both phases of system response (shift of *SWT *and onset of graded control) emerge again, but the graded control strategy is achieved earlier for lower environmental variance than in the case of equally distributed season length (Fig. 4 and 5). This is not surprising, as the normal distribution is not bounded and even allows for season lengths of only one day. The normal distribution is characterised by very smooth slopes on both flanks of the distribution. The rather smooth increase of the probability density on the left flank is reflected in a smooth increase in sexual production. In contrast to the case of the uniform distribution the strategy transition is thus nearly monotonic (Fig. 4f, in contrast to Fig. 2f).

The field observations of colony dynamics of *L. malachurum *yielded a rather broad transition zone with a period of approximately 11 days with simultaneous production of workers and sexuals ([24] and unpublished). For realistic standard deviations of season length of about 5 days this transition zone is far too broad to be explained as a bet-hedging strategy. According to our model, bet-hedging would predict a pure bang-bang strategy for such a standard deviation under a uniform distribution of season length (Fig. 3), and only a transition phase of less than 5 days under the assumption of a normal distribution in season length (Fig. 5).

## Discussion

Our analysis of the optimal resource allocation pattern in eusocial insect colonies clearly demonstrates that moderate fluctuations of environmental conditions (length of foraging season) will not necessarily foster the evolution of bet-hedging allocation strategies. This deviation from the rather intuitive predictions of Oster and Wilson is readily explained by the inherent buffering capacity of the bang-bang allocation strategy; finite worker productivity and mortality rates determine an extended reproductive phase at the end of the season, when sexuals are produced exclusively. Thus, even if season length would be rather short, colonies could nonetheless expect certain fitness (successful production of sexuals) as long as the season ends after the onset of the reproductive phase. Consequently, rather high fluctuations in environmental conditions are needed to promote the evolution of graded allocation strategies with the simultaneous production of workers and sexuals.

The results of our model are rather robust against variation in model parameters (worker mortality, worker survival and mean season length). Parameter modifications within a plausible range did not change results markedly. It seems plausible to assume that increasing mean season length might reduce the effect of environmental variance, as identical environmental variance decreases relatively when mean season length increases. This is not the case. Increasing mean season length will just prolong the period of complete worker production, but not influence strategy transition. Even more, very short mean season length could result in strategies which start with the production of sexuals right from the beginning [33,46-48].

As long as a season ends after the onset of the reproductive phase the pure bang-bang strategy is buffered against complete reproductive failure. The switch from the bang-bang strategy to a graded strategy thus strongly depends on the length of the reproductive phase. Worker efficiency and survival are the main determinants of the switching time in the deterministic case without environmental fluctuations and the optimal duration of sexual production decreases with increasing worker survival (*q*) and increasing worker efficiency (*c*) [8]. Populations in ideal conditions with high worker survival and high worker efficiency will thus switch to graded allocation strategies for much smaller variability of environmental conditions than populations that live under harsh environmental conditions.

Yet, at least for *Lasioglossum malachurum *the broad transition zone between pure colony growth and reproduction cannot be explained as an adaptive response to fluctuating environmental conditions. Even for normally distributed season length the observed transition zone is more than twice as long as predicted based on realistic estimates for the coefficients of variation for environmental fluctuations. Model results for uniformly distributed season length indicate that with more realistic distribution functions and observed variability of season length graded control is rather improbable.

According to our model, a transition phase between worker and sexual production is not necessarily characterised by a smooth continuous (sigmoid) increase in the production of sexuals as has been predicted by Oster and Wilson [11]. The specific form of this transition strongly depends on the frequency distribution of season length. For uniformly distributed season length with a very steep left flank, the transition zone is characterised by a peak in the amount of resources invested into reproduction of sexuals at the beginning of the transition phase. On the other hand, for the normal distribution, with its very smooth left flank the transition is characterised by a monotonic increase in the amount of resources invested into reproduction.

**Figure 6:**
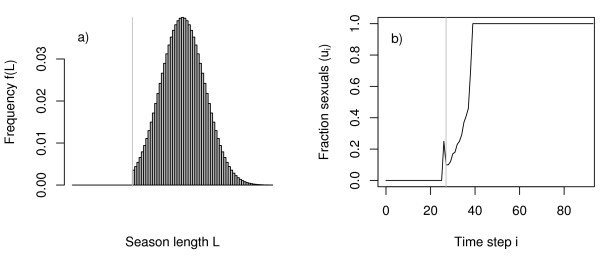
Optimal investment strategy (a), when 1% of the frequency distribution of season length is cut at the left side of the distribution (b). There is a prominent peak in sexual production just before minimum season length. Model parameters: worker productivity rate *c *= 0.15, survival rate *q *= 0.95, mean season length *μ *= 50, standard deviation *σ *= 8.7.

We have chosen the rectangular and the normal distribution because they are both commonly used types of frequency distributions representing the opposite ends of a continuum of distributions with increasingly steep flanks [30,33]. However, the normal distribution is an unbounded distribution while the minimum as well as the maximum length of a season is obviously bounded. Thus, assuming a normal distribution of season length may easily produce artefacts in the context of bet hedging. This can be seen, when the variance of season length is greatly increased. As we have to limit season length to a minimum of one day an increase in the variance of the normal distribution necessarily leads to steeper left flanks of this distribution. When we do this, the peak in resource allocation observed in the case of the uniform distribution emerges again. An increase of the lower boundary of the season length (in Fig. 3 and Fig. 5 we assume a minimum seasons length of one day, *L*_*min *_= 1) will also reestablish the humped shape for predicted resource allocation. A lower boundary of *L*_*min *_= 27 (about half of the mean season length) which cuts only 1% of the normal distribution in Fig. 4c will result in a prominent peak in the strategy curve. Immediately after the onset of the reproductive phase nearly 30% of the resources are invested into the production of sexuals. This rather high investment decreases subsequently to values around 15% before it rises again to end with a pure sexual phase (Fig. 6). In general this hump becomes more pronounced when either the length of the season becomes more variable or when the left flank of the density distribution of the season length becomes steeper (Fig. 2).

We have shown that variation in season length is not likely to be the reason for graded control in halictids. We have given two arguments. In general, environmental variation can already be buffered by simple bang-bang strategies. Parameter and effect estimation for the sample species *L. malachurum *in fact lead to the conclusion that the environmental variation this species is exposed to is too low to necessitate graded control as an evolutionary answer.

Instead, we believe that alternative mechanisms can be responsible for the evolution of graded control in this species. For the sake of simplicity (and to accord with the simple model by Macevicz and Oster) we have assumed constant productivity and mortality rates during the season. However, it is reasonable that worker (per capita) productivity declines, when colony size increases. That this in fact occurs has been shown by Michener for several halictid species [49]. Although different theoretical approaches tried to analyse the effect of decreasing worker productivity within the framework of optimal dynamic resource allocation, the most straight forward analysis has never been performed: replacing the linear dependence of resource allocation on worker number (see eqns. 1 and 2) by a logistic relationship. Surprisingly, Macevicz and Oster [8] used such a relationship only for the worker equation (see eqn. 1 here) to estimate model parameters from field data, but not for the dynamics of the sexuals, thus inevitably favouring the early production of sexuals. Their remark, that saturation of productivity can promote graded control only for very restrictive parameter combinations seems premature, and further theoretical effort on this topic should be promising. Beekman *et al.*[9] investigated the effect of limited egg laying rate in bumble bees. This is equivalent to saturation of colony productivity, but unfortunately they allowed only for dichotomous strategy switches in their model, i.e. did not foresee the evolution of graded control. In this case the evolution of early switching is always accompanied by a waste of time. As the egg laying rate of *L. malachurum *queens is limited, too [35], this may be a reason for the graded allocation strategy observed in this species. First model calculations (unpublished) support this hypothesis. Thus, a thorough analysis should incorporate a broader set of strategic options to predict the influence of rate limitations on resource allocation strategies. However, it has to be kept in mind that workers of *L. malachurum *are not sterile. Thus, the queen's limited egg laying rate does not necessarily imply a saturation of egg production, as workers might provide additional eggs [35].

The original model of Macevicz and Oster [8] as well as our approach consider the colony (homologous to a single individual) as the unit of selection. As long as we assume that only a single trait is variable (and independent from others) and that all individuals of the colony do not have any further behavioural options, this perspective is also valid at the individual and the genetic level. However, since Macevicz and Oster there has been a wealth of theoretical and empirical analyses on individual worker fitness and the balance between gene, individual and colony level selection (see [50] for review). Selfishness, e.g. a worker disappearing into hibernation to become a queen the following year or leaving the colony for independent nest founding, may also account for 'graded control', even though this may be sub-optimal at the level of the colony. For most eusocial halictids, workers probably have a variety of reproductive options ( see [51] for cases of 'worker-sized' queens in *L. malachurum *that enter hibernation to found colonies the following year). The same may be true for social polistine wasps, where the distinction between gyne and worker toward the end of the colony cycle (and even earlier in the colony cycle) is difficult [52]. The timing of the production of males is another aspect that has benefited from a gene-centred analysis [53].

## Conclusion

Up to now bet-hedging as an evolutionary response to variation in season length has been the main argument to explain field observations of graded resource allocation in annual eusocial insects. However, our analysis shows that the effect of moderate environmental fluctuations does not select for deviation from the classical bang-bang strategy and that the evolution of graded allocation strategies can be triggered only by extreme fluctuations. Thus, the widespread belief that graded control in social insects is most probably a type of spreading of risk was premature.

Both, additional behavioural mechanisms at the colony level and gene-centred or individual-centred approaches can provide promising alternative explanations. Detailed quantitative field or laboratory observations on resource allocation in eusocial insects are required to analyse the relevance of alternative explanations, e.g. logistic colony growth or reproductive conflict between queen and workers, for the evolution of graded allocation strategies.

## Methods

The numerical solution of optimal control problems with objective functions averaging over different realisations (here: different season length) of the dynamic system (here eqns. 1 and 2) in a nonlinear way (here: geometric mean) cannot be achieved with standard dynamic programming [54]. However, nesting the dynamic programming approach within a recurrence procedure is a suitable way to find the optimal control function, if the iteration converges.

We first focus on the longest season length *L*_*max *_possible with respect to the distribution of season length. The optimal value *u*_*Lmax *_can be chosen independently from state and control function at earlier time steps, just based on maximising *S*_*Lmax*. _Working backwards (dynamic programming) in time requires re-definition of the objective function. To find *u*_*Lmax*-1 _we have to maximise *S*_*Lmax *_· *S*_*Lmax*-1_. This expression can be expanded in terms of state and control function values in time step *L*_*max*-1_. However, in general (for this and earlier time steps) it is not possible to find the value of *u*_*i *_that maximises this expression without knowing the numbers of queens and workers in the current time step. Furthermore these values cannot be determined without knowing the values of *u*_*i *_in previous time steps. This circularity can be broken by an iterative procedure. We first assume a trial solution *u*_i_* for the control function and calculate the numbers of queens and workers in different time steps. Then an approximate value for *u*_*i *_(not optimal yet) can be determined assuming the numbers of queens and workers just calculated. The approximate value can be calculated by derivating the objective function with respect to *u*_*i *_symbolically and finding the root numerically. This procedure is repeated for all time steps back to *i *= 0. This yields an approximate solution for *u*_*i *_(*i *= 0, ..., *L*_*max*_).

Now the numbers of queens and workers can be recalculated using the approximate values of *u*_*i *_from above. Next the optimal values of *u*_*i *_can be recalculated, too. This process is repeated until the values of *u*_*i *_have converged to the required accuracy (changes in the Euclidian norm of (*u*_1_, .., *u*_*Lmax*_) less than 0.001).

There might also be an analytical solution of the optimal control problem based on the application of Pontryagin's Maximum Principle, but we did not follow this line of approach ([33] provides detailed instructions).

## Authors' contributions

All authors participated in the design of the study. OM developed the mathematical model, implemented and carried out the numerical calculations and drafted the manuscript. NW provided and presented the field data. ES and H-JP conceived of the study, analysed and interpreted the numerical results and helped to draft the manuscript. All authors read and approved the final manuscript.

## References

[B1] Roff DA (1992). The evolution of life histories.

[B2] Stearns S (1992). The evolution of life histories.

[B3] Cohen D (1971). Maximizing final yield when growth is limited by time or by limiting resources. Journal of Theoretical Biology.

[B4] Perrin N, Sibly RM (1993). Dynamic models of energy allocation and investment. Annual Review of Ecology and Systematics.

[B5] Iwasa Y (2000). Dynamic optimization of plant growth. Evolutionary Ecology Research.

[B6] Cole LC (1954). The population consequences of life history phenomena. Quarterly Review of Biology.

[B7] Kozlowski J, Teriokhin AT (1999). Allocation of energy between growth and reproduction: The Pontryagin Maximum Principle solution for the case of age- and season dependent mortality. Evolutionary Ecology Research.

[B8] Macevicz S, Oster GF (1976). Modeling social insect populations II: Optimal reproductive strategies in annual eusocial insect colonies. Behavioral Ecology and Sociobiology.

[B9] Beekman M, Lingeman R, Kleijne FM, Sabelis MW (1998). Optimal timing of the production of sexuals in bumblebee colonies. Entomologia Experimentalis et Applicata.

[B10] Mitesser O, Weissel N, Strohm E, Poethke HJ (2006). The evolution of activity breaks in the nest cycle of annual eusocial bees: a model of delayed exponential growth. BMC Evol Biol.

[B11] Oster GF, Wilson EO (1978). Caste and ecology in the social insects.

[B12] Michener CD (2000). The bees of the world.

[B13] Greene A, Akre RD, Landolt P (1976). The aerial yellowjacket, *Dolichovespula arenaria *(Fab.): nesting biology, reproductive production, and behavior (Hymenoptera: Vespidae). Melanderia.

[B14] Smith FK (1956). A colony of yellow jackets, *Vespula pennsylvanica*. Entomology News.

[B15] Blackith RE, Stevenson JH (1958). Autumnal populations of wasps nests. Insectes Sociaux.

[B16] Haggard CM, Gamboa GJ (1980). Seasonal variation in body size and reproductive condition of a paper wasp, *Polistes metricus *(Hymenoptera: Vespidae). Canadian Entomologist.

[B17] Greene A (1984). Production schedules of vespine wasps: an empirical test of the bang-bang optimization model. Journal of the Kansas Entomological Society.

[B18] Kolmes SA (1986). Have hymenopteran societies evolved to be ergonomically effcient?. Journal of the New York Entomological Society.

[B19] Martin SJ (1991). A simulation model for colony development of the hornet *Vespa simillima *(Hymenoptera, Vespidae). Japanese Journal of Entomology.

[B20] Roseler P (1970). Unterschiede in der Kastendetermination zwischen den Hummelarten *Bombus hypnorum *und *Bombus terrestris*. Zeitschrift für Naturforschung.

[B21] Müller CB, Schmid-Hempel P (1992). Variation in life-history pattern in relation to worker mortality in the bumblebee *Bombus lucorum*. Functional Ecology.

[B22] Yanega D (1988). Social plasticity and early-diapausing females in a primitively social bee. Proceedings of the National Academy of Sciences of the United States of America.

[B23] Yanega D (1993). Environmental inuences on male production and social structure in *Halictus rubicundus *(Hymenoptera: Halictidae). Insectes Sociaux.

[B24] Strohm E, Bordon-Hauser A (2003). Advantages and disadvantages of large colony size in a halictid bee: the queen's perspective. Behavioral Ecology and Sociobiology.

[B25] Hirata M, Cronin AL, Kidokoro M, Azuma N (2005). Spatio-temporal variation of colony structure and eusociality level of the Japanese sweat bee *Lasioglossum (Evylaeus) duplex *(Hymenoptera : Halictidae). Ecological Research.

[B26] Weissel N, Mitesser O, Poethke HJ, Strohm E (2006). The influence of soil temperature on the nesting cycle of the halictid bee *Lasioglossum malachurum*. Insectes sociaux.

[B27] Menu F, Roebuck JP, Viala M (2000). Bet-hedging diapause strategies in stochastic environments. The American Naturalist.

[B28] Menu F, Desouhant E (2002). Bet-hedging for variability in life cycle duration: bigger and later-emerging chestnut weevils have increased probability of a prolonged diapause. Oecologia.

[B29] Kozlowski J, Ziolko M (1988). Gradual transition from vegetative to reproductive growth is optimal when the maximum rate of reproductive growth is limited. Theoretical population biology.

[B30] Bradford MJ, Roff DA (1997). An empirical model of diapause strategies of the cricket *Allonemobius Socius*. Ecology.

[B31] Kaitala V, Mappes TY, loenen H (1997). Delayed female reproduction in equilibrium and chaotic populations. Evolutionary Ecology.

[B32] Hopper KR (1999). Risk-spreading and bet-hedging in insect population biology. Annual Review of Entomology.

[B33] King D, Roughgarden J (1982). Graded allocation between vegetative and reproductive growth for annual plants in growing seasons of random length. Theoretical Population Biology.

[B34] McNamara JM (1994). Timing of entry into diapause: Optimal allocation to growth and reproduction in a stochastic environment. Journal of Theoretical Biology.

[B35] Knerer G (1992). The biology and social behaviour of *Evylaeus malachurus *(K.) (Hymenoptera; Halictidae) in different climatic regions of Europe. Zoologische Jahrbücher: Abteilung für Systematik, Ökologie und Geographie der Tiere.

[B36] Sakagami SF Sozialpolymorphismus bei Insekten Probleme der Kastenbildung im Tierreich.

[B37] Yodiz P (1989). Introduction to theoretical ecology.

[B38] Bradford MJ, Roff DA (1993). Bet hedging and the diapause strategies of the cricket *A llonemobius fasciatus*. Ecology.

[B39] Wong TG, Ackerly DD (2005). Optimal reproductive allocation in annuals and an informational constraint on plasticity. The New Phytologist.

[B40] Taylor BE, Gabriel W (1993). Optimal adult growth of *Daphnia *in a seasonal environment. Functional Ecology.

[B41] Wolfram Research Inc (1999). Mathematica, Vers 40.

[B42] Ihaka R, Gentleman R (1996). R: A language for data analysis and graphics. Journal of Computational and Graphical Statistics.

[B43] Mitesser O, Strohm E, Weissel N, Poethke HJ (2007). Optimal resource allocation in primitively eusocial bees: a balance model based on investment limitation of the queen. Insectes sociaux.

[B44] Legewie H (1925). Zum Problem des tierischen Parasitismus, I. Teil: Die Lebensweise der Schmarotzerbiene *S phecodes monilicornis K. (= subquadratus) *(Hymenoptera: Apoidea). Zeitschrift für Morphologie und Ökologie der Tiere.

[B45] Schmid-Hempel P, Durrer S (1991). Parasites, oral resources and reproduction in natural populations of bumblebees. Oikos.

[B46] Sakagami SF, Munakata M (1972). Distribution and bionomics of a transpalearctic eusocial halictine bee *Lasioglossum evylaeus calceatum *in northern Japan with reference to its solitary life cycle at high altitude. Journal of the Faculty of Science Hokkaido University Series VI Zoology.

[B47] Eickwort GC, Eickwort JM, Gordon J, Eickwort MA (1996). Solitary behavior in a high altitude population of the social sweat bee *Halictus rubicundus *(Hymenoptera: Halictidae). Behavioral Ecology and Sociobiology.

[B48] Packer L, Jessome V, Lockerbie C, Sampson B (1989). The phenology and social biology of four sweat bees in a marginal environment Cape Breton Island Nova Scotia Canada. Canadian Journal of Zoology.

[B49] Michener CD (1964). R eproductive effciency in relation to colony size in hymenopterous societies. Insectes Sociaux.

[B50] Ratnieks FLW, Foster KR, Wenseleers T (2006). Conflict resolution in insect societies. Annual Review of Entomology.

[B51] Richards MH, French D, Paxton RJ (2005). It's good to be queen: classically eusocial colony structure and low worker fitness in an obligately social sweat bee. Molecular Ecology.

[B52] Reeve HK The social biology of wasps.

[B53] Fletcher DJC, Ross KG (1985). Regulation of reproduction in eusocial Hymenoptera. Annual Review Of Entomology.

[B54] Mangel M, Clark CW (1989). Dynamic modeling in behavioral ecology.

